# Dengue epidemic typology and risk factors for extensive epidemic in Amazonas state, Brazil, 2010–2011

**DOI:** 10.1186/s12889-018-5251-x

**Published:** 2018-03-15

**Authors:** Daniel Barros de Castro, Vanderson Souza Sampaio, Bernardino Cláudio de Albuquerque, Rosemary Costa Pinto, Megumi Sadahiro, Ricardo Augusto dos Passos, Cristiano Fernandes da Costa, José Ueleres Braga

**Affiliations:** 1 Fundação de Vigilância em Saúde do Amazonas, Manaus, Brazil; 2 Escola Nacional de Saúde Pública Sérgio Arouca – Fiocruz, Rio de Janeiro, Brazil; 3 Fundação de Medicina Tropical Dr Heitor Vieira Dourado – FMT-HVD, Manaus, Brazil; 4 Instituto Oswaldo Cruz – Fiocruz, Rio de Janeiro, Brazil; 5 Instituto de Medicina Social – UERJ, Rio de Janeiro, Brazil; 6 PECTI-SAÚDE / Fundação de Amparo a Pesquisa do Estado do Amazonas, Manaus, Brazil

**Keywords:** Dengue epidemics classification, Socioeconomic, Environment, Climate, Amazon

## Abstract

**Background:**

Dengue is the most prevalent arboviral disease affecting humans. The frequency and magnitude of dengue epidemic have significantly increased over recent decades. This study aimed to identify dengue epidemic types and risk factors for the extensive epidemics that occurred in 2010–2011, across the municipalities of Amazonas state, Brazil.

**Methods:**

Using an ecological approach, secondary data were obtained from the dengue fever surveillance system. Epidemic waves were classified according to three indices: duration, intensity, and coverage. A hierarchical model of multiple logistic regression was used for the identification of risk factors, with the occurrence of extensive dengue epidemic.

**Results:**

During the study period, dengue virus affected 49 of the 62 Amazonas municipalities. In 22 of these, the epidemics were of high intensity, wide range, and long time span, and therefore categorized as “extensive epidemics”. The final multivariable model revealed a significant association between extensive dengue epidemics occurrence and the average number of days with precipitation (adjusted OR = 1.40, 95% CI: 1.01–1.94) and the number of years with infestation (adjusted OR = 1.53, 95% CI: 1.18–1.98).

**Conclusions:**

Our results indicate that it is crucial to integrate vector control, case management, epidemiological investigation, and health education, in order to respond to the growing threat of multiple mosquito-borne diseases, such as dengue, Zika and chikungunya, which are highly prevalent in the South America region.

**Electronic supplementary material:**

The online version of this article (10.1186/s12889-018-5251-x) contains supplementary material, which is available to authorized users.

## Background

Dengue is a acute febrile disease, transmitted by arthropod vectors, and caused by four different virus (DENV-1, DENV-2, DENV-3, DENV-4). Dengue virus infections may be asymptomatic or lead to a range of clinical presentations, even death [1]. The clinical manifestations include acute fever, frontal headache, retroocular pain, muscle and joint pain, nausea, vomiting, and rash [2, 3]. The illness, generally, is self-limited and lasts approximately for one week. Patients may eventually develop severe disease, characterized by acute fever with minor or major bleeding, evidence of plasma leakage, and organ involvement [4].

Dengue infections occur in more than 100 countries, reaching approximately 50 million cases each year [5]. In the Americas, the occurrence of recurring dengue outbreaks every 3–5 years with an increasing number of cases over time shows the transition from an endemic-epidemic state to a highly endemic state in recent years [6]. In the state of Amazonas, Brazil, although there is a high density of mosquitoes throughout the seasons, there is usually a peak of the disease in the first half of the year [7–9]. Since 1998, when the dengue virus was first detected in Manaus, Amazonas state capital, an increasing number of countryside municipalities have been affected by dengue epidemics. During 2010 and 2011, dengue disease affected 49 of the 62 municipalities in Amazonas [9].

Dengue fever has been shown to spread by epidemic waves, that is, the number of reported cases suddenly increases until reaching a peak and then gradually decreases until the epidemic ends [10]. However, the magnitude of dengue epidemics is variable. Epidemics appear to largely reflect susceptibility of the human population to circulating serotypes and mosquito density; however, they are also affected by climatic, environmental, meteorological, social and demographic conditions [11, 12].

Hopp and Foley [13] built a model based on climatic variables in order to predict the mosquito population size, which was related to the number of dengue cases in South Asia and Central America. Hales et al. [14] investigated the role of meteorological covariates in determining the pattern of spatial distribution of dengue cases, concluding that average vapor pressure was the main predictor of dengue distribution. According to the authors, the average vapor pressure, which is a measure of humidity, is high only where rainfall and temperatures are high, and these are conditions that are conducive to breeding and survival of vector populations, and rapid replication of the virus [14].

In addition to recognizing epidemics, it is important to be aware of epidemic magnitude, as milder epidemics have less damaging consequences for the population and their control can be achieved with less complex measures. Conversely, extensive epidemics are more difficult to control, entails a greater financial burden and cause more damage to the population, due to the increased mortality rate and the occurrence of severe cases and temporary disability which lead to significant economic losses [14].

Although much is known about climatic and environmental conditions (temperature and precipitation) and population characteristics (vulnerability to circulating subtypes) associated with epidemic occurrence [6], there is a lack of knowledge about the conditions that determine the large scale, extent and duration of epidemics, that is, the causes of “extensive dengue epidemics”.

Wen et al. [15] criticized the use of dengue annual incidence as an only measure of epidemic wave intensity and justified the adoption of different measures to characterize epidemic severity. The authors suggested three relevant indicators: (i) frequency index, defined as the number of weeks with recorded cases divided by the total number of weeks of the study period; (ii) duration index, defined as the number of weeks with recorded cases divided by the number of epidemic waves; (iii) intensity index, the incidence rate divided by the number of epidemic waves.

The recognition of factors (modifiable and uncontrollable) associated with extensive epidemics is fundamental for the adoption of measures aimed at reducing modifiable causes. Furthermore, the identification of extensive epidemic-likely scenarios would enable control program intensification or the implementation of different strategies to mitigate the effects of the epidemic.

## Methods

This study aimed: (i) to identify dengue epidemic types, and (ii) risk factors for extensive epidemics occurring in 2010–2011 in the municipalities of Amazonas state, Brazil.

**Fig. 1:**
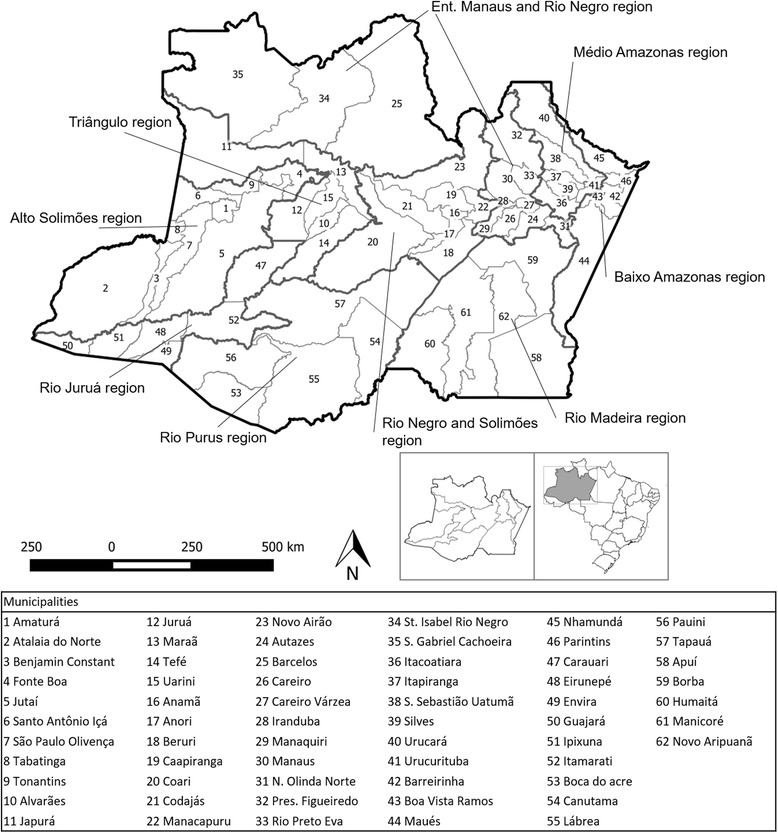
Municipalities and health regions of the state of Amazonas

The state of Amazonas is located in Northern Brazil and is the largest federative unit, with an area of 1,559,161 km^2^, divided into 62 municipalities and 9 health regions (Fig. 1). It counts 3,483,985 inhabitants, of whom 79% lived in urban zones, in 2010. Approximately 52% (1,802,525 inhabitants) of the state’s population resided in the capital Manaus [16]. The general population density in the state was 2.2 inhabitants / km^2^ and 158 inhabitants / km^2^ in the capital [16]. The predominant climate is equatorial, characterized by high temperatures and high rainfall indices [17].

We conducted an ecological study using secondary data from dengue fever surveillance system. Amazonas state municipalities and calendar months were considered as the units of analysis.

Data on dengue cases were extracted from the Notifiable Diseases Information System (called SINAN) provided by the Health Surveillance Foundation (called FVS-AM) of the Health Department of the State of Amazonas. We included all records of dengue cases in SINAN, reported between January 1 2001, and December 31 2011, defined as classical dengue, dengue with complications, dengue hemorrhagic fever or dengue shock syndrome. The classification of the cases followed the criteria established by the Ministry of Health, in accordance with the World Health Organization (WHO) recommendations [18]. Dengue cases were georeferenced by the municipality of patient residence.

The population resident in the municipalities and regions was obtained from the censuses conducted by the Brazilian Institute of Geography and Statistics (IBGE) in 2000 and 2010. From these data, population estimates for the inter-census years were calculated using a linear interpolation technique [19].

### Dengue epidemic typology and extensive epidemic definition

The identification of epidemic periods was performed based on analysis of statistical process control charts prepared for each municipality. The control charts were constructed according to the following steps: (i) calculation of the average monthly incidence rate for the period 2001 to 2010; (ii) visual inspection of the monthly epidemic curve of dengue disease in each municipality to identify putative epidemic months (PEM) with a substantial increase in incidence rates; (iii) determination of the epidemic months, defined by an epidemic onset (the PEM with a positive change in the incidence rate greater than 60% of the incidence of the preceding month) and a termination point (the PEM with a negative change in the incidence rate greater than 60% of the incidence of the preceding month; (iv) excluding periods (epidemic months) with high incidence rates; (v) calculation of the expected maximum incidence rate corresponding to the sum of the average incidence rate with 1.96 the standard deviation of this rate; (vi) identification of the epidemic months, characterized by a monthly incidence rate in 2010 and 2011 exceeding the expected maximum limit of the control chart.

An epidemic wave was defined as a set of consecutive epidemic months (more than 1 month) when the epidemic occurred in a given municipality or the epidemic lasting 1 month with incidence greater than 10 cases / 100 thousand inhabitants. In this study, we propose a typology of epidemic that includes two measures used in the method described by Wen et al. [15] in combination with a third aspect relating to the spatial extent of the epidemic. We defined the extent of the epidemic wave based on the variation of incidence rates calculated for each month, consistently with the method reported by Parker and Holman [11]. Epidemic waves were classified according to three indices: duration, intensity, and coverage.

The duration index α was calculated by the ratio of the sum of epidemic months (EM) divided by the number of epidemic waves (EW) observed in each municipality, where i indicates the index of the epidemic wave.

$$ \alpha =\frac{\sum_1^i EM}{nEW} $$

The intensity index ß was calculated by the ratio of the sum of the average monthly incidence rates (IR) of each epidemic wave and the number of epidemic waves (EW) observed in each municipality, where i indicates the epidemic wave index.

$$ \beta =\frac{\sum_1^i IR}{nEW}. $$

The spatial coverage index Υ was calculated by the ratio of the number of municipalities with epidemic waves (MEW) in a given region and the total number of municipalities in the region (TMR).

$$ Y=\frac{nMEW}{nTMR} $$

According to the variation of the values of each index, three categories were defined and a score ranging from 1 to 3 was assigned (Table 1).

**Table 1:** Scores according to criteria categories for epidemics classification

Criterion	Feature	Definition	Score
Duration (based on index α)	Low	Up to 3 epidemic months	1
	Middle	With 4 to 6 epidemic months	2
	High	Greater than 6 epidemic months.	3
Intensity (based on the ß index)	Low	Incidence rates in epidemic months less than 10 cases / 100 thousand inhabitants.	1
	Middle	Incidence rates in epidemic months between 10^a^ and 100 cases / 100 thousand inhabitants.	2
	High	Incidence rates in epidemic months above 100^b^ cases / 100 thousand inhabitants	3
Coverage (based on index Υ)	Limited	Up to 1/3 of the municipalities in the region presented epidemic months	1
	Moderated	From 1/3 and 2/3 of the municipalities in the region presented epidemic months	2
	Wide	Greater than 2/3 of the municipalities in the region had epidemic months	3

^a^1st quartile of distribution of dengue incidence rates; ^b^3th quartile of distribution of dengue incidence rates

From the sum of the scores for each criterion, a global score was calculated for each municipality, ranging from 1 to 9. Ultimately, the municipalities with an overall score of 8 or higher were considered with “extensive dengue epidemics” (EDE).

### Study variables

The following socioeconomic information was examined: illiteracy rate, municipal human development index (HDI), Gini index of income, average household income per capita, proportion of the poor population, unemployment rate of the population aged 18 or over, proportion of population in households with bathroom and piped water, proportion of population in households with garbage collection, and proportion of urban population. These indicators were obtained from the 2010 demographic census. The definition and calculation of these indicators is detailed in Additional file 1: Table S1.

The meteorological variables used were: wind speed, insolation, days with precipitation, precipitation, minimum temperature, maximum temperature, relative humidity, and compensated temperature. The river quota parameters analyzed were: maximum quota, minimum quota, and average quota. The meteorological and river quota variables analyzed refer to the period from January 2010 to December 2011, same period that extensive dengue epidemics were studied. See detailed information on obtaining these indicators in Additional file 1: Table S2.

Vector control actions were measured by Stegomyia indices widely used in the scientific literature [20], which are: average annual proportion of home visit targets achieved; number of years with an achieved target of 5 home visits; number of years with infestation; appropriate ratio of number of agents per building; appropriate ratio of number of supervisors by health agent; adequate ration of supervisors and agents per building; household infestation mean index for the period; and number of years with household infestation mean index greater than 1%. These indicators refer to annual averages recorded between 2010 and 2011. The vector control activities data were obtained from the FVS’s Department of Environmental Surveillance and Disease Control. See detailed information on obtaining these indicators in Additional file 1: Table S3.

### Statistical analysis

We performed an analysis of completeness and inconsistencies in the dengue database. Records with similarities regarding patient name, date of birth, and mother’s name, and those with a period of less than 60 days between reporting dates were identified as duplicate records, and were excluded.

Summary measures (mean, standard deviation, minimum and maximum values) were used to describe the distribution of study variables. A hierarchical model of multiple logistic regression was used for the identification of risk factors, with the occurrence of extensive dengue epidemic, defined above, as the as the outcome variable. Associations between socioeconomic, meteorological, and performance characteristics of dengue surveillance services in the municipalities of Amazonas and the outcome (EDE) were initially analyzed separately for each group (hierarchical level). Subsequently, for the construction of the final model, we selected the explanatory variables showing an association with the outcome for a level of significance of 0.20 by stepwise technique backward mode. Variables associated with the outcome at a significance level of 0.05 remained in the final model. The multicollinearity between the explanatory variables selected for the final model was analyzed using the variance inflation factor test, and those variables with VIF greater than 10 were considered collinear and excluded of the model. Crude and adjusted odds ratios of the association between the outcome and the explanatory variables were calculated. Statistical package STATA v.13 (StataCorp, 2013, College Station, Texas, USA) was used to perform these analyzes.

## Results

Between 2001 and 2011, 85,216 cases of dengue fever were recorded in the Amazonas state. However, between 2010 and 2011, the largest epidemic occurred, resulting in 58,296 reported cases involving 49 (80%) municipalities in the state.

**Fig. 2:**
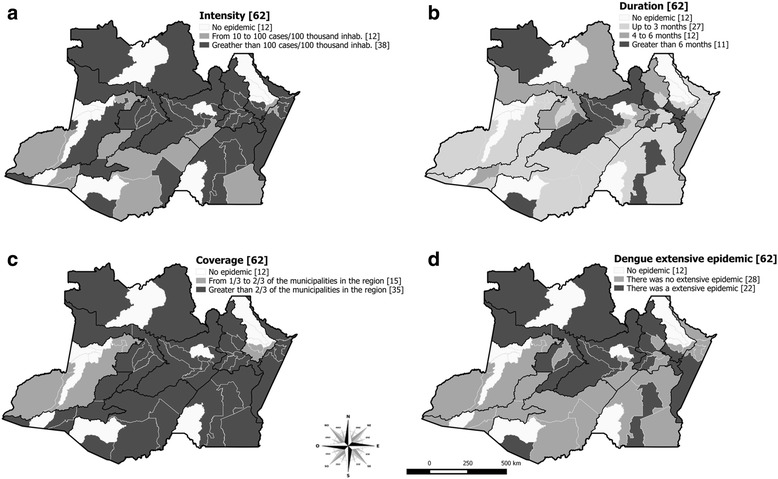
Spatial distribution of classification dimensions of dengue epidemics and extensive epidemic by Amazonas Municipalities in 2011. **a** Intensity. **b** Duration. **c** Coverage. **d** Extensive dengue epidemics

During this epidemic, most of the municipalities recorded at least one monthly dengue incidence rate greater than 100 cases per 100,000 inhabitants (Fig. 2a) over at least one month. With the exception of the Alto Solimões and Médio Amazonas regions, in all regions 2/3 or more of the municipalities were affected by the dengue epidemic (Fig. 2a-c). Extensive epidemics were observed in 35% (22/62) of municipalities, mostly located in Entorno de Manaus and Rio Negro region (55% [11/20]) (Fig. 2d).

The socioeconomic and demographic profile of amazon municipalities shows a high average illiteracy rate (21%), high percentage of poor population (53%), and low human development index (0.56). The most populated municipalities are Manaus, with 1.8 million inhabitants in 2010, and Parintins, with 101 thousand inhabitants. The other municipalities have less than 90 thousand inhabitants. Manaus has the highest population concentration in urban areas, with 99% of the urban population. In Parintins, the urban population corresponds to 68% of the total population. The meteorological feature results indicated a low wind speed (0.9 km/h) and high precipitation rate (215.3 mm^3^). The river quota recorded in the municipalities presented a considerable variation, ranging from a minimum average quota of 316 cm to a maximum average quota of 2653 cm. The municipalities of Amazonas are characterized by a high household vector infestation and an insufficient number of health agents and supervisors. Generally, the less populous municipalities present a recent history of *Aedes* infestation and lower average rates of infestation (Table 2).

**Table 2:** Socioeconomic profile, meteorological findings, river quota, and operational characteristics of dengue surveillance services in Amazonas municipalities

Feature	N	Mean	SD	Max.	Min.
Socioeconomic profile					
Illiteracy rate	62	20.9	9.7	40.7	4.0
Income Gini Index	62	0.6	0.1	0.8	0.5
Proportion of poor	62	53.0	12.0	74.2	12.9
Per capita income	62	251.3	98.3	790.3	122.2
Unemployment rate	62	7.6	3.3	18.9	1.6
Prop. of pop. in households with bathroom and piped water	62	36.2	16.0	83.6	9.5
Prop. of pop. in households with garbage collection	62	80.3	19.8	100.0	0.1
Human Development Index (HDI)	62	0.56	0.1	0.7	0.4
Proportion of urban population	62	54.9	14.3	99.5	4.2
Meteorological and river quota					
Average wind speed	62	0.9	0.4	1.8	0.1
Annual average heat stroke	62	156.0	19.8	193.5	121.9
Average days with precipitation	62	15.8	1.7	18.9	12.6
Annual average rainfall	62	215.3	23.5	253.5	167.9
Average minimum temperature	62	23.2	0.9	25.0	21.4
Average maximum temperature	62	32.8	0.4	33.8	32.1
Average relative humidity	62	84.5	2.1	87.3	79.8
Average temperature compensated	62	27.0	0.5	28.0	26.1
Average of the maximum quota	62	1181.0	470.0	2653.0	425.2
Average of the minimum quota	62	960.7	469.8	2482.5	316.2
Average of the middle quota	62	1068.9	469.3	2553.2	382.6
Vector Control					
Proportion of goals reached from home visits	62	26.1	29.3	88.1	0
Number of years with a target of 5 home visits reached ^a^	62	0.4	0.8	3	0
Number of years with infestation ^a^	62	2.2	2.4	5	0
Appropriate ratio of the number of agents per property	62	0.3	0.5	1	0
Appropriate ratio of the number of supervisors to health agent	62	0.6	0.5	1	0
Adequacy of the number of supervisors and agents for amount of building	62	1.1	1.4	3	0
Household infestation mean index for the period	62	0.5	0.9	3.6	0
Number of years with household infestation mean index higher than 1%	62	0.6	1.1	4	0

^a^Recorded between 2006 and 2010

The proportion of poor, per capita income, and proportion of the population in households with bathroom/ piped water were associated with the extensive dengue epidemics. When considering this level alone, an increase of one unit in per capita income increased the risk of severe epidemic occurrence by 1% (adjusted OR = 1.1, 95% CI: 1.0–1.1), after controlling for the other variables of this level (Table 3).

**Table 3:** Socioeconomic factors associated with extensive dengue epidemics using bivariate and multivariable hierarchical level analysis

	Crude OR (95% CI)	*p*-value	Adjusted OR (95% CI) - Hierarchical levels	*p*-value
Illiteracy rate	0.9 (0.9–1.0)	0.151	0.9 (0.8–1.1)	0.202
Gini Index	0.5 (0–3848.1)	0.868	–	–
Proportion of poor	0.9 (0.9–1.0)	0.023	1.0 (0.9–1.2)	0.771
Per capita income	1.0 (1.0–1.1)	0.011	1.0 (1.0–1.1)	0.011
Unemployment rate	1.1 (0.9–1.3)	0.211	–	–
Proportion of population in households with bathroom and piped water	1.0 (1.0–1.1)	0.022	1.0 (0.9–1.1)	0.610
Proportion of population in households with garbage collection	1.0 (0.9–1.1)	0.106	1.0 (0.9–1.1)	0.440
HDI	1.0 (0.3–3.5)	0.831	1.0 (0.1–8.5)	0.065
Proportion of urban population	0.9 (0.9–1.0)	0.174	0.9 (0.9–1.1)	0.380

*HDI* Human Development Index, *OR* Odds ratio

No meteorological and hydrology hierarchical level factors were significantly associated with EDE occurrence. However, the average number of days with precipitation, when adjusted for other conditions, increased the risk of EDE (adjusted OR = 1.4, 95% CI: 1.1–1.9), (Table 4).

**Table 4:** Meteorological and hydrological factors associated with extensive dengue epidemic using bivariate and multivariable hierarchical level analysis

	Crude OR (95% CI)	p-value	Adjusted OR (95%CI) - Hierarchical levels	*p*-value
Average wind speed	2.9 (0.8–10.2)	0.092	31.7 (0.15–6521.4)	0.092
Annual average heat stroke	1.0 (1.0–1.1)	0.052	0.9 (0.8–1.1)	0.486
Average days with precipitation	1.4 (0.9–2.0)	0.057	1.4 (1.1–1.9)	0.018
Annual average rainfall	1.0 (0.9–1.0)	0.993	–	–
Average minimum temperature	1.6 (0.8–2.9)	0.137	0.1 (0.1–3.7)	0.159
Average maximum temperature	0.9 (0.2–3.5)	0.875	–	–
Average relative humidity	0.9 (0.7–1.2)	0.640	–	–
Average compensated temperature	2.2 (0.8–6.1)	0.133	5477.3 (0.5–5555.6)	0.064
Average of the maximum quota	1.0 (1.0–1.1)	0.052	1.5 (0.2–15.8)	0.741
Average of the minimum quota	1.0 (1.0–1.1)	0.052	1.1 (1.0–1.2)	0.144
Average of the middle quota	1.0 (1.0–1.1)	0.052	6.85 (0.75–62.98)	0.089

The operational level model showed the number of years with infestation, adequate ratio of number of agents per property, adequate ration of supervisors and agents per building, household infestation mean index for the period, and number of years with household infestation mean index greater than 1% as predictors of EDE. Controlling for confounders, the risk of EDE increased by 54% with each increase by one unit in the number of years with infestation (adjusted OR = 1.5, 95% CI: 1.2–2.0), (Table 5).

**Table 5:** Vector control activities associated with extensive dengue epidemic using bivariate and multiple logistic regression analysis

	Crude Odds Ratio (95% CI)	*p*-value	Adjusted Odds Ratio (95% CI)	*p*-value
Average proportion of goals reached from home visits	1.0 (1.0–1.1)	0.121	0.9 (0.9–1.0)	0.100
Number of years with an achieved target of 5 home visits	1.8 (0.9–3.5)	0.075	3.5 (0.8–15.7)	0.107
Number of years with infestation	1.5 (1.2–2.0)	0.001	1.5 (1.2–2.0)	0.001
Appropriate ratio of the number of agents per property	4.8 (1.5–15.1)	0.007	495.8 (0.1–1260.0)	0.410
Appropriate ratio of the number of supervisors to health agent	2.7 (0.9–8.2)	0.087	0.3 (0.1–3.9)	0.352
Adequate ration of supervisors and agents per building	1.8 (1.2–2.7)	0.004	0.1 (0.1–63.9)	0.520
Household infestation mean index for the period	2.3 (1.2–4.5)	0.013	1.6 (0.3–7.2)	0.567
Number of years with household infestation mean index greater than 1%	1.8 (1.1–3.1)	0.020	1.8 (0.5–5.9)	0.355

The final multivariable model revealed a significant association between EDE occurrence and the average number of days with precipitation and the number of years with infestation. The risk of EDE increased by 40% as the average number of days with precipitation increased by one unit (adjusted OR = 1.4, 95% CI: 1.1–1.9). Moreover, the risk increased by 53% as the number of years with infestation increased by one unit (adjusted OR = 1.5, 95% CI: 1.2–2.0) when controlling for the average number of days with precipitation (Table 6).

**Table 6:** Extensive dengue epidemic predictors based on hierarchical multiple logistic regression analysis

	Adjusted Odds Ratio (95% CI)	*p*-value
Average number of days with precipitation	1.4 (1.1–1.9)	0.041
Number of years with infestation	1.5 (1.2–2.0)	0.009

## Discussion

In areas where dengue is considered endemic, it is likely that larger epidemics may play a relevant role in the spread of disease and health status of these populations. Here, three features of the epidemics were evaluated in order to classify them according to epidemic dimensions. Only the larger epidemics, classified as extensive epidemics, were evaluated in this study to examine epidemic distribution and associated factors. Several authors use epidemic metrics to characterize dengue outbreaks [11, 12, 15, 21]. According to Galli and Chiaravalloti Neto [22], the use of these epidemic indices as an alternative to the use of the incidence rate allows the identification of areas and periods of greatest risk.

Among the 49 municipalities that recorded dengue cases in the study period, we identified 22 municipalities that met the criteria for extensive dengue epidemics, that is, this municipalities presented epidemics with high intensity, extent, and duration. These municipalities surround the Capital and the main economical centers of the State, including Coari, Tefé, and Parintins. Extensive dengue epidemics occurrence in these municipalities is likely to support the widespread dispersal of dengue cases in the region, revealing the potential for dissemination of other arboviruses, such as Zika and Chikungunya, as well as the complexity of controlling these diseases in the region. In areas with widespread disease distribution, it is important to differentiate the severity levels of the areas affected by the disease. This approach allows investigate with greater precision the etiological factors of large epidemics, and also it is an important tool for public managers and decision makers, since they generate alerts with different risk levels.

Multivariable analysis results indicate that climatic and environmental conditions, as well as vector control activities, has independent effects on occurrence of extensive dengue epidemics in the Amazonas municipalities.

The urbanization process triggered by economic development may favor the establishment and spread of the *Aedes* mosquito, due to its ability to adapt to urban environments [21]. Furthermore, population mobility facilitates viral circulation and the climate changes generated in this space enhance disease transmission cycle [23]. Our results corroborate this hypothesis as the risk of extensive dengue epidemics is higher in the municipalities with higher economic development, characterized by higher average per capita income, a larger proportion of the population living in households with toilets and water supply, and lower proportion of poor population. Even when controlling for other socioeconomic conditions, average per capita income was associated with the occurrence of extensive epidemics.

Seasonal climatic forecasts are important tools for dengue epidemics prediction [24, 25]. The findings of this study show that an increase in insolation time, number of days with precipitation, and river quota, increased the risk of extensive dengue epidemics occurrence, presenting borderline *p*-value in the single regression model. Cazelles et al., studied synchronous dengue epidemics in 2005 and found a positive correlation between the incidence of dengue hemorrhagic fever and temperature and precipitation increase [26]. Other studies also showed a positive association between these climate variables and river height with dengue [25, 27], demonstrating the consistency of our findings.

The National Dengue Control Programme in Brazil [18] advocates home visits to 100% of the properties every two months and, during epidemic periods, intensification of vector control actions in locations previously determined by the LIRAa (vector infestation rate surveys). Our results showed that municipalities with dengue epidemics had insufficient number of agents and supervisors and a high household infestation index. These findings support the hypothesis that structural problems in public health services, such as insufficient numbers of health agents, can lead to the non-execution of or failure to achieve target home visits, and, consequently, the increase of vector infestation rates in a given locality. As quoted by Gubler [28], most mosquito control actions focused on eliminating adult vectors, with an unsuitable method and, without changing people’s lifestyles, they continue to provide habitats larvae in their homes. This may result in the vector’s geographical expansion and increased mosquito population density, increased viral circulation and occurrence of cases and severe epidemics.

Precipitation and non-effective control measures were the best predictors of extensive epidemic risk, when considering proximal and distal levels. In our model, socioeconomic conditions may be understood as distal variables, influencing outcome to a lesser extent than the proximal ones, explaining why they were not included in the final model. This corroborates the results of other studies identifying an association between dengue epidemics and rainfall and household vector infestation [29–32]. Furthermore, socioeconomic factors (per capita income), other environmental factors (river water level) and other vector control measures adopted (adequacy of human resources for vector control level of building infestation) can also identify municipalities with a high propensity to extensive epidemics.

Secondary data obtained from the SINAN database were used in this study. There are generally issues with the underreporting, completeness and quality of this type of data. However, in this study only variables with at least 70% of completeness were considered. Searches for duplicates records were performed and dates were reviewed to identify any mistakes.

## Conclusions

The findings presented here showed influences of local and regional climate variability on the occurrence of extensive dengue epidemics. These results can provide basic knowledge for the development of an early warning system in the future and support for decision makers implementing dengue prevention strategies under different environmental conditions. Furthermore, there is a strong need to integrate vector control, case management, epidemiological investigation, and health education, in order to respond to the growing threat of multiple mosquito-borne diseases, such as dengue, Zika, and chikungunya, highly prevalent in South American.

## Additional file

Additional file 1: Structure of epidemic indicators, Epidemic indicators definition, units and scale. (DOCX 21 kb)

## Abbreviations

CI: Confidence interval
EDE: Extensive dengue epidemic
EM: Epidemic months
EW: Epidemic wave
FVS-AM: Health Surveillance Foundation
HDI: Human development index
IR: Incidence rate
Max: Maximum value
MEW: Municipalities with epidemic waves
Min: Minimum value
N: Sample size
OR: Odds ratio
PEM: Putative epidemic months
SD: Standard deviation
SINAN: Notifiable Diseases Information System
TMR: Total number of municipalities in the region
WHO: World Health Organization
