# Supplementary material for: Dengue epidemic typology and risk factors for extensive epidemic in Amazonas state, Brazil, 2010–2011
Source: BMC Public Health. 2018 Mar 15;18:356. doi: 10.1186/s12889-018-5251-x (PMC5855995; doi:10.1186/s12889-018-5251-x)
Supplement: Supplementary file 1 — Structure of epidemic indicators, Epidemic indicators definition, units and scale. (DOCX 21 kb) [file 12889_2018_5251_MOESM1_ESM.docx]

**Additional file 1**

Structure of epidemic indicators.

Table S1. Socioeconomic indicators.

| **Indicator** | **Unit/Scale** | **Definition/** **Calculation** |
| --- | --- | --- |
| Illiteracy rate of the population aged 18 years or over | % | The ratio between the population 18 years of age or older who cannot read or write a simple message and the total number of people in this age group, multiplied by 100. |
| Gini index of per capita household income | NA* | Measures the degree of inequality existing in the distribution of individuals according to the per capita household income. Its value is 0 when there is equality (i.e., the per capita household income of all individuals has the same value) and tends towards 1 as the inequality increases. The considered individuals were limited to those living in permanent private households. |
| Proportion of the poor population | % | The proportion of individuals with a per capita household income equal to or less than R$255.00 per month in August 2010, which equivalent to 1/2 the minimum wage on that date. The considered individuals were limited to those living in private households. |
| Household income per capita | R$ (reais) | Household income per capita is calculated by dividing the household income equally by the total number of residents. |
| Unemployment rate of population aged 18 years or over | % | Proportion of economically active population in this age group who was unemployed, (i.e., those who were not employed in the week prior to the date of the census but who had sought work over the month prior to the date of this study). |
| Proportion of population in households with bathroom and piped water | % | It is calculated by dividing the number of people living in households with water supply from the general network and whose sanitary sewage is carried out by sewage collection system or septic tank and the total population living in permanent private households, multiplied by 100. |
| Proportion of population in households with garbage collection | % | It is calculated by dividing the number of people living in households with garbage collection and the total population living in permanent private households, multiplied by 100. |
| Municipal Human Development Index | NA* | Geometric mean of the indices of the Income, Education and Longevity dimensions, with equal weights. |
| Proportion of urban population | % | It is calculated by dividing the resident population in urban area by the total population, multiplied by 100. |

* NA: not applicable

Table S2. River quota and meteorological indicators.

| **Indicator** | **Unit/Scale** | **Definition/** **Calculation** |
| --- | --- | --- |
| Wind speed | Meters per second | Monthly average wind speed. |
| Insolation | Hours | Monthly average heat stroke. |
| Days with precipitation | Number of days | Monthly average days with precipitation. |
| Precipitation | Millimeters | Monthly average rainfall. |
| Minimum temperature | °C | Monthly average minimum temperature. |
| Maximum temperature | °C | Monthly average maximum temperature. |
| Relative humidity | °C | Monthly average relative humidity. |
| Compensated temperature | °C | Monthly average temperature compensated. |
| Maximum quota | Centimeters | Monthly average of the maximum quota. |
| Minimum quota | Centimeters | Monthly average of the minimum quota. |
| Average quota | Centimeters | Monthly average of the middle quota. |

Table S3. Vector control indicators.

| **Indicator** | **Unit/Scale** | **Definition/** **Calculation** |
| --- | --- | --- |
| Number of years with a target of 5 home visits reached | Years | This indicator was calculated by adding the number of years with an achieved target of 5 home visits. Recorded between 2006 and 2010. |
| Proportion of goals reached from home visits | % | This indicator was calculated by dividing the number of home visit carried out by number of home visit expected. |
| Number of years with infestation | Years | Number of years with *Aedes* infestation. Recorded between 2006 and 2010. |
| Appropriate ratio of the number of agents per property | Agents/ building | For each year studied was assigned the value equal to 1 if the municipality had at least 1 agent per 1,000 building and value equal to 0 if this ratio was less than 1 agent per 1,000 building. This indicator was calculated by the annual average (from 2010 and 2011) that the municipality presented an adequate number of agents per building. |
| Appropriate ratio of the number of supervisors to health agent | Supervisors/agents | For each year studied was assigned the value equal to 1 if the municipality had at least 1 supervisor per 10 health agents and value equal to 0 if this ratio was less than 1 supervisor per 10 agents. This indicator was calculated by the annual average (from 2010 and 2011) that the municipality presented an adequate number of supervisor per agent. |
| Adequacy of the number of supervisors and agents for amount of building | NA* | For each year studied the value was assigned equal to 0 if the municipality presented an insufficient number of agents and supervisors; Value equal to 1 if the municipality presented adequate number of supervisors, but not of agents; Value equal to 2 if the number of agents was adequate, but not that of supervisors; And value equal to 3 if the number of agents and supervisors were adequate. This indicator was calculated by the annual average (from 2010 and 2011) that the municipality presented an adequate number of agents or supervisors. |
| Household infestation mean index for the period | % | Annual mean of the proportion of positive residences for *Aedes* larvae. |
| Number of years with household infestation mean index higher than 1% | Years | Number of years with household infestation mean index greater than 1%. |

* NA: not applicable
